# Morbidity associated with emergency surgery *versus* scheduled surgery in patients with placenta accreta spectrum

**DOI:** 10.61622/rbgo/2024rbgo74

**Published:** 2024-09-06

**Authors:** Juan José Saldarriaga-Hoyos, Daniela Sarria-Ortiz, Valentina Galindo-Velasco, Luisa Fernanda Rivera-Torres, Albaro José Nieto-Calvache

**Affiliations:** 1 Fundación Valle del Lili Departamento de Ginecología y Obstetricia Cali Colombia Fundación Valle del Lili, Departamento de Ginecología y Obstetricia, Cali, Colombia.; 2 Universidad Icesi Facultad de Ciencias de la Salud Cali Colombia Universidad Icesi, Facultad de Ciencias de la Salud, Calle, Cali, Colombia.; 3 Fundación Valle del Lili Centro de Investigaciones Clínicas Cali Colombia Fundación Valle del Lili, Centro de Investigaciones Clínicas, Cali, Colombia.

**Keywords:** Placenta accreta, Morbidity, Emergencies, Obstetric surgical procedures

## Abstract

**Objective:**

This study aims to evaluate the clinical outcomes of surgical management for placenta accreta spectrum in a Latin American reference hospital specializing in this condition. The evaluation involves a comparison between surgeries performed on an emergent and scheduled basis.

**Methods:**

A retrospective cohort study was conducted on patients with placenta accreta spectrum who underwent surgery between January 2011 and November 2021 at a hospital in Colombia, using data from the institutional PAS registry. The study included patients with intraoperative and/or histological confirmation of PAS, regardless of prenatal suspicion. Clinical outcomes were compared between patients who had emergent surgeries and those who had scheduled surgeries. Descriptive analysis involved summary measures and the Shapiro-Wilk test for quantitative variables, with comparisons made using Pearson’s Chi-squared test and the Wilcoxon rank sum test, applying a significance level of 5%.

**Results:**

A total of 113 patients were included, 84 (74.3%) of them underwent scheduled surgery, and 29 (25.6%) underwent emergency surgery. The emergency surgery group required more transfusions (72.4% vs 48.8%, p=0.047). Patients with intraoperative diagnosis of placenta accreta spectrum (21 women, 19.5%) had a greater volume of blood loss than patients taken into surgery with known presence of placenta accreta spectrum (3500 ml, IQR 1700 – 4000 vs 1700 ml, IQR 1195-2135. p <0.001).

**Conclusion:**

Patients with placenta accreta spectrum undergoing emergency surgery require transfusions more frequently than those undergoing scheduled surgery

## Introduction

Placenta accreta spectrum (PAS) is associated with maternal morbidity, particularly postpartum hemorrhage, and urinary tract injuries.^1^The incidence of PAS is increasing due to the rising rates of cesarean sections.^2^Diagnose PAS is challenging and requires active searching, especially in patients with coexistence of placenta previa and a history of one or more cesarean sections. Prenatal diagnosis is desirable, as it enables patients to be referred to hospitals with adequate resources and trained interdisciplinary teams.^3^

It has been reported that patients undergoing scheduled surgery, following the prenatal diagnosis of this condition, experience less bleeding, fewer transfusions, and fewer urinary tract injuries than those undergoing emergency surgery.^4-7^However, other patient series managed at PAS reference centers do not find differences in outcomes when comparing scheduled and emergency surgeries.^8,9^The possibility of unexpected bleeding in the presence of placenta previa and PAS motivates the programmed termination of gestation between 35 and 36 weeks, despite a higher likelihood of requiring neonatal support due to prematurity.^3,10,11^

The objective of this study is to compare clinical outcomes between emergency and scheduled surgeries in PAS patients in a PAS referral center in a middle-income country.

## Methods

A retrospective cohort study was conducted, including patients with PAS who underwent surgical management between January 2011 and November 2021 at a designated PAS reference hospital in Colombia. Data were taken from the institutional PAS registry.^12^All patients with intraoperative and/or histological confirmation of PAS, with or without prenatal suspicion (based on ultrasound and/or magnetic resonance imaging), were included. Emergency surgery was defined as interventions performed before the scheduled date determined by the PAS team, within the first 6 hours after hospital admission due to vaginal bleeding or other medical condition. All other patients were considered to have undergone scheduled surgery. Clinical outcomes were compared between patients undergoing emergency and scheduled surgery (asymptomatic patients undergoing surgery at the planned time by the interdisciplinary team).

Additionally, a comparison of outcomes was conducted between patients in whom the diagnosis of PAS was known before cesarean section based on ultrasound or magnetic resonance imaging (MRI) results, and those without prenatal suspicion of PAS (with unexpected intraoperative diagnosis).

The surgical management protocol for PAS at Fundación Valle de Lili, Cali, Colombia, has been previously described.^12,13^It involves the participation of an interdisciplinary team with experience in dealing with PAS. When the diagnosis of PAS arises unexpectedly during a cesarean section prompted by another obstetric condition (intraoperative finding of PAS), the attending surgeons immediately notify the PAS team who respond within less than 30 minutes. Patients with prenatal suspicion of PAS are admitted to the hospital one day before surgery, which is performed in the 35th week of gestation. Intraoperative bleeding is objectively measured by adding the blood volume from suction devices to that from surgical drapes and compresses.

Descriptive analysis was performed using summary measures and dispersion for quantitative variables, according to the normality of their distribution, which was assessed using the Shapiro-Wilk test. Absolute frequencies and percentages were calculated for qualitative variables. Pearson’s Chi-squared test was used to compare proportions in two independent populations, and the Wilcoxon rank sum test was used for the comparison of quantitative variables that did not follow a normal distribution. A significant level of 5% was employed for all tests. The statistical analysis was conducted using R software version 4.2.1 (R Foundation for Statistical Computing) through RStudio 2022.12.0.

The study was approved by the medical ethics committee of Fundación Valle del Lili (Protocol No. 2034; Minutes No. 01-2023). Informed consent from the patients was not obtained due to the retrospective nature of the study and the anonymized presentation of data.

## Results

The study included 113 patients, with an average age of 32 years, and 83.2% of them had a history of previous uterine surgery. Demographic characteristics are summarized in table 1. 84 patients underwent elective surgeries (74.3% of the sample), while 29 patients underwent emergency surgical procedures (25.6% of the sample). Notably, the group undergoing emergency surgery required blood transfusions at a significantly higher frequency (72.4% versus 48.8%, p=0.047) (Table 2).

**Table 1:** Demographic and clinical characteristics of women with Placenta Accreta Spectrum

Variables		n(%)
Age^*^		32.6(5.7)
Pregnancies	1	5(4.4)
	2-3	69(61.1)
	>3	39(34.5)
Previous cesarean sections	0	20(17.7)
	1-2	83(73.5)
	>2	10(8.8)
Previous uterine surgery	No	19(16.8)
	Cesarean section	93(82.3)
	Dilation and Curettage	4(3.5)
Timing of the diagnosis	Preoperative	91(80.5)
	Intraoperative	22(19.5)
Gestational age (trimester) at the Time of PAS Diagnosis	First trimester (≤12 weeks)	3(2.7)
	Second trimester (13 to 28 weeks)	42(37.2)
	Third trimester (≥29 weeks)	68(60,2)
Degree of PAS According to FIGO Classification**	1	48(42.5)
	2	36(31.9)
	3a	12(10.6)
	3b	11(9.7)
	3c	6(5.3)

^*^Mean (SD); ^**^Jauniaux et al. (2019);^(14)^ PAS - placenta accreta spectrum

**Table 2:** Clinical outcomes of women with Placenta Accreta Spectrum according to the urgency of surgery

Variables	Type of surgery		p-value^**^
	Scheduled (n = 84) n(%)	Emergency (n = 29) n(%)	
Bleeding in surgery^*^	1800mL (1233mL-2500mL)	1700mL (1200mL-2500mL)	0.6
Transfusion of RBCU	41(48.8)	21(72.4)	0.047
Admission to intensive care unit	64(76.2)	26(89.7)	0.2
Blader injury	15(17.9)	8(27.6)	0.4
Ureteral Injury	7(8.3)	1(3.4)	0.6

*Median (IQR); **Wilcoxon rank sum test; Pearson’s Chi-squared test; PAS - placenta accreta spectrum; RBCU - red blood cell units

Table 3 outlines clinical outcomes when comparing patients with prenatal suspicion of PAS (91 patients, 80.5%) and those without (22 patients, 19.5%). Patients without prenatal suspicion of PAS experienced a higher volume of blood loss (3500 ml, IQR 1700-4000) compared to those diagnosed with PAS prenatally (1700 ml, IQR 1195-2135). Among patients without a prenatal diagnosis of PAS, there was a trend towards a higher need for blood transfusion and a greater incidence of ureteral injuries, although statistical significance was not reached.

**Table 3:** Clinical outcomes of women with Placenta Accreta Spectrum according to the timing of diagnosis

Variables	Timing of PAS diagnosis		p-value^**^
	Preoperative (n = 91) n(%)	Intraoperative (n = 22) n(%)	
Bleeding in surgery^*^	1700mL (1195mL-2135mL)	3500mL (1700mL-4000mL)	<0.001
Transfusion of RBCU	46(50.5)	16(72.7)	0.10
Admission to intensive care unit	72(79.1)	18(81.8)	>0.9
Blader Injury	18(19.8)	5(22.7)	>0.9
Ureteral Injury	5(5.5)	3(13.6)	0.4

*Median (IQR); n(%); **Wilcoxon rank sum test; Pearson’s Chi-squared test; PAS - Placenta Accreta spectrum; RBCU - red blood cell units

## Discussion

Patients with PAS undergoing surgery with limited interdisciplinary team preparation, either due to an emergency or lack of preoperative PAS diagnosis, exhibit inferior clinical outcomes compared to those undergoing scheduled surgery led by a specialized PAS team. Prenatal diagnosis of PAS allows for improved planning and coordination of the surgical approach.^15^Scheduled surgeries conducted by a dedicated PAS team typically result in improved outcomes due to the availability of appropriate resources, expertise, and careful preoperative planning.^16^These factors contribute to the mitigation of complications.

A higher frequency of blood transfusions was observed in patients undergoing emergency surgery (Table 2), and a greater volume of intraoperative bleeding was noted in women with a PAS intraoperative diagnosis (Table 3). The most consistently associated factor with improved clinical outcomes in PAS cases is the involvement of interdisciplinary teams in hospitals equipped with adequate resources.^16^Some research groups, even in emergency situations, have reported comparable outcomes between patients with and without prenatal diagnoses.^8,9^This similarity in outcomes may be attributed to the fact that, even in emergency situations, patients receive care from experienced healthcare professionals. The presence of an interdisciplinary team specializing in PAS management can significantly impact the quality of care and, consequently, patient outcomes. However, it’s noteworthy that these specialized teams may not always be readily available.

The availability of interdisciplinary teams regularly exposed to a high number of PAS cases and equipped with the recommended resources for managing this condition is limited in low and middle-income countries (LMICs).^17^ Our results illustrate that even in a hospital serving as a reference center for PAS, patients who undergo surgery without a prior PAS diagnosis or as an emergency procedure shortly after hospital admission (due to vaginal bleeding) experience higher blood loss and more frequent blood transfusions. While our observation did not specifically evaluate whether initial care was provided by the PAS team of our hospital or by personnel without specific training in PAS management, it is reasonable to assume that, in cases without prenatal suspicion, the PAS team may not have been involved from the outset of the surgery.

Other series in LMICs also concur in highlighting that patients undergoing scheduled surgery experience lower blood loss^18^and require fewer blood transfusions.^19^This observation is consistent with findings from high-income countries.^6^

This study has limitations that should be considered when interpreting its findings. The challenge of defining parameters to distinguish patients undergoing surgery under controlled conditions (scheduled surgery with a prior Placenta Accreta Spectrum (PAS) diagnosis) from other scenarios, characterized by varying degrees of urgency, different amounts of vaginal bleeding, and variable availability of human and hospital resources, is indeed formidable. Our definition of emergency surgery (surgery within 6 hours of admission) attempts to encompass patients requiring immediate surgical intervention, such as those with vaginal bleeding, preterm labor, or fetal distress. We consider this definition as valid, particularly in our hospital, where most patients scheduled for surgery are admitted the day before their procedure. However, these considerations may vary across different hospitals.

For this reason, we conducted an additional analysis comparing patients with intraoperative diagnoses of PAS, who unequivocally require emergent care. In both comparisons, patients for whom the surgical team had more time to prepare exhibited better outcomes. Despite the entire population being treated in the same hospital, with access to technological resources (interventional radiology, cell saver, aortic occlusion devices, advanced bipolar energy, etc.), multiple medical specialties (urologists, vascular surgeons, gynecologic oncologists, etc.), and where a PAS team is available 24 hours a day within 30 minutes after an emergency call, our findings suggest that the critical factor in the clinical outcome of PAS patients is the use of an appropriate surgical technique from the beginning of the surgery by personnel with specific training in disease management, beyond their academic background. This aligns with previous studies indicating that poorer clinical outcomes are primarily associated with the use of inappropriate surgical techniques and the involvement of surgeons without the necessary training.^20^

The retrospective nature of this study and the convenience sample size suggest that it is important to validate our observations through additional studies. Nonetheless, PAS is a relatively rare condition, and this study is one of the largest series reported in Latin America. While it is plausible that scheduled procedures and prenatal diagnosis facilitate the involvement of PAS teams in the surgical management of PAS, potentially explaining our results, there are multiple uncontrolled variables that may influence the associations proposed in this study, thus limiting the external validity of our findings.

The results of this study emphasize the need of improve responses to intraoperative discoveries of PAS or the emergent presentation of patients with a known PAS diagnosis. Of particular importance is the development of a clear guide delineating which interventions to perform and which to omit for general obstetricians encountering this condition unexpectedly during the execution of a cesarean section prompted by another obstetric condition. Further research and efforts to enhance the management of PAS in various clinical settings are warranted to validate and extend the insights provided by this study.

## Conclusion

Patients with PAS undergoing emergency surgery require blood transfusions more frequently than those with scheduled procedures. Similarly, patients diagnosed intraoperatively with PAS experience greater intraoperative blood loss compared to those with a preoperative diagnosis. It is likely that an elective scenario, where the prenatal diagnosis is known, facilitates the preparation and involvement of interdisciplinary teams dedicated to managing PAS.
